# Early classical swine fever virus persistence is associated with cytotoxic and immunoregulatory transcriptional signatures distinct from protective C-strain vaccination

**DOI:** 10.3389/fcimb.2026.1956070

**Published:** 2026-09-24

**Authors:** Sara Puente-Marin, Adriana Muñoz-Aguilera, Anibal Asilvera, Cristina Riquelme, Liani Coronado, Yoandry Hinojosa, Saray Heredia, Àlex Cobos, Llilianne Ganges

**Affiliations:** 1 WOAH Reference Laboratory for Classical Swine Fever, IRTA-CReSA, Barcelona, Spain; 2 Unitat mixta d’Investigacio´ IRTA-UAB en Sanitat Animal, Centre de Recerca en Sanitat Animal, Bellaterra, Barcelona, Spain; 3 IRTA, Programa de Sanitat Animal, Centre de Recerca en Sanitat Animal, Bellaterra, Barcelona, Spain; 4 Subgerencia Análisis y Diagnóstico, Instituto Colombiano Agropecuario, Bogotá, Colombia; 5 Departament de Sanitat i Anatomia, Animals, Facultat de Veterinària, Universitat Autònoma de Barcelona, Barcelona, Spain

**Keywords:** CSFV, cytotoxic response, interferon response, PBMCs, persistent infection, RNA sequencing

## Abstract

Persistent classical swine fever virus (CSFV) infection represents a major challenge for disease control because persistently infected pigs remain clinically healthy while continuously shedding virus. However, the early immunological events associated with the establishment of viral persistence remain incompletely understood and require further investigation. In this study, we compared the peripheral blood mononuclear cell (PBMC) transcriptomes of neonatal piglets infected with the moderately virulent Catalonia01 CSFV strain, previously shown to induce postnatal persistent infection, with those of piglets vaccinated with the live attenuated C-strain vaccine. Samples were collected at 7 days post-infection or vaccination, before the onset of clinical signs or detectable anti-CSFV antibodies. Catalonia01-infected piglets exhibited high viral RNA loads and increased serum IFN-α levels, accompanied by enrichment of JAK–STAT and cytokine signaling pathways, indicating robust early innate antiviral activation. Transcriptomic analysis also revealed a marked cytotoxic transcriptional signature together with increased expression of immunoregulatory genes and lower expression of genes associated with MHC class II-mediated antigen presentation relative to C-strain-vaccinated animals. Together, these findings indicate that the early establishment of persistent infection occurs despite a robust antiviral transcriptional response, suggesting that immune activation alone is insufficient to achieve effective viral control. Collectively, these findings reveal distinct early transcriptional programs associated with Catalonia01 infection and protective C-strain vaccination, with persistent infection characterized by concurrent antiviral activation and immunoregulatory transcriptional signatures, further supporting and expanding the emerging concept of complex immune regulation during CSFV persistence.

## 1 Introduction

Classical swine fever (CSF) remains one of the most important transboundary viral diseases affecting swine worldwide and continues to represent a major threat to animal health, food security, and the global pig industry. Due to its severe economic impact, rapid spread, and implications for international trade, CSF is a notifiable disease to the World Organisation for Animal Health (WOAH). The etiological agent, classical swine fever virus (CSFV), infects both domestic and wild suids and can induce a wide spectrum of disease outcomes ranging from acute lethal disease to chronic, subclinical, congenital, and persistent infections depending on viral virulence, host age, and immune status (Muñoz-González et al., 2015b; Ganges et al., 2020; World Organisation for Animal Health (WOAH), 2025). Among the different clinical presentations of CSF, persistent infection represents one of the greatest challenges for disease control and eradication. Besides congenital persistence following transplacental infection, previous studies demonstrated that postnatal infection of neonatal piglets with moderately virulent CSFV strains can also generate persistent infection (Muñoz-González et al., 2015b). Persistently infected animals may remain clinically healthy for prolonged periods despite maintaining extremely high viral loads in blood, tissues, and secretions, including nasal and rectal excretions, thereby acting as highly efficient reservoirs for silent viral dissemination (Muñoz-González et al., 2015b; Bohorquez et al., 2019). Importantly, these animals fail to mount effective humoral and cellular immune responses against CSFV, remain seronegative for E2- and E^rns^-specific antibodies, do not develop neutralizing antibodies, and are unable to respond efficiently to vaccination (Muñoz-González et al., 2015b; Muñoz-González et al., 2015a; Bohorquez et al., 2019). Consequently, their detection under field conditions is particularly difficult, especially in endemic settings where surveillance frequently relies on serology-based approaches (Coronado et al., 2019).

Previous studies demonstrated that persistently CSFV-infected piglets exhibit defective IFN-γ responses in peripheral blood mononuclear cells (PBMCs), increased IL-10 production, impaired adaptive immune responses, and expansion of immature granulocytic 6D10^+^ cell populations displaying immunosuppressive characteristics compatible with myeloid-derived suppressor cells (MDSCs) (Muñoz-González et al., 2015b; Bohórquez et al., 2019; Bohorquez et al., 2019). Moreover, these animals progressively lose the capacity to mount efficient type I interferon responses during later stages of infection, even following exposure to unrelated highly immunostimulatory viruses such as African swine fever virus (ASFV), suggesting the establishment of profound systemic immune dysregulation favoring viral adaptation and persistence (Cabezón et al., 2017). Altogether, these observations suggest that persistent CSFV infection involves immune reprogramming beyond passive immune evasion in the context of long-term viral replication. This highlights the need to further characterize the early immune events associated with the establishment of persistence.

The ability of viruses to establish persistence through modulation of host immune responses is a common feature shared by several chronic viral infections affecting both humans and animals. Persistent infections caused by viruses such as hepatitis B virus (HBV), hepatitis C virus (HCV), human immunodeficiency virus (HIV), lymphocytic choriomeningitis virus (LCMV), and herpesviruses are characterized by sustained inflammatory signaling, progressive dysfunction of antiviral immunity, chronic antigenic stimulation, and induction of immunoregulatory pathways that ultimately impair viral clearance (Odorizzi and Wherry, 2013; Snell et al., 2017; Teer et al., 2025). In these settings, persistent viral infections are frequently associated with progressive immune dysfunction, including activation of immunoregulatory pathways and impaired antiviral T-cell responses (Kahan et al., 2015; Belk et al., 2022). Similar mechanisms have also been associated with defective antigen presentation and impaired development of protective adaptive immunity (Zuniga et al., 2015). Therefore, understanding the early molecular events distinguishing protective from dysfunctional antiviral responses has relevance not only for CSFV pathogenesis but also for persistent viral infections more broadly. Recent transcriptomic studies have provided important insights into host immune responses during CSFV infection, particularly regarding dendritic cell activation, innate immune sensing, and virulence-associated inflammatory responses (Auray et al., 2020). In addition, highly virulent CSFV strains were associated with stronger inflammatory and antiviral transcriptional responses together with impaired antigen presentation functions in dendritic cells (Coronado et al., 2025). However, these studies have primarily focused on acute infection or *in vitro* models, whereas the early systemic molecular mechanisms underlying postnatal persistent infection in neonatal piglets remain largely unexplored. Likewise, comparative transcriptomic analysis between persistently infected and vaccinated animals during the same early stage are still lacking.

In the present study, we performed transcriptomic profiling of PBMCs from neonatal piglets infected with the moderately virulent CSFV Catalonia01 strain, previously characterized by its ability to induce postnatal persistent infection, and compared their responses with those of piglets vaccinated with the live attenuated C-strain vaccine at 7 days post-infection or post-vaccination. At this early point, animals remained clinically healthy and seronegative, providing an opportunity to examine transcriptional differences before the development of overt disease or detectable humoral responses. The aim of this study was to characterize and compare the early transcriptional programs associated with Catalonia01 infection leading to persistence and protective C-strain vaccination. Within this comparative framework, we sought to identify molecular signatures distinguishing these two divergent early immune outcomes that may provide insight into the host response associated with the establishment of persistent CSFV infection.

## 2 Materials and methods

### 2.1 Cells and viruses

The porcine kidney cell line PK-15 (ATCC-CCL-33) was grown in Eagle’s Minimum Essential Medium supplemented with 5% fetal bovine serum (FBS) and was used for viral production and titration. The CSFV Catalonia01 strain (genotype 2.3) and the C-strain vaccine (Pestiffa^®^) was used for *in vivo* experiments.

### 2.2 Experimental design

Four pregnant Pestivirus-free sows (Landrace) of 100 days into gestation were housed in the Animal Biosafety Level 3 (ABSL3) facility at IRTA-CReSA (Barcelona, Spain) and randomly allocated into two groups (2 sows per group). The sows were housed in a box with standard facilities to allocate pregnancy and lactating sows. Their farrowings were synchronized with 75 µg d-cloprostenol/sow at 114 days of gestation. One sow in group 2 and her litter was euthanized immediately after parturition due to welfare concerns and were excluded from subsequent analysis.

After 6 days after birth, piglets in group 1 were inoculated intranasally with 1 mL of 2.5 x 10^4^ TCID_50_ of CSFV Catalonia01 strain. After 3 days of birth, piglets in group 2 were inoculated intranasally with 100 PD_50_ per dose of C-strain vaccine, as recommended by manufacturer. The piglets remained with their mothers throughout the experiment. After viral or vaccine inoculation, daily clinical monitoring, including rectal temperature, was carried out by a trained veterinarian in a blinded manner. In accordance with previous studies (Lamothe-Reyes et al., 2021), a clinical score value from 0 to 6 was assigned daily to each animal: 0, no signs; 1, mild fever; 2, mild to moderate clinical signs; 3, moderate clinical signs; 4, moderate to severe clinical signs; 5, severe clinical signs; and 6, death. Serum and nasal and rectal swab samples as well as blood samples for the isolation of peripheral blood mononuclear cells (PBMCs) were collected at 7 days post-inoculation (dpi) or vaccination (dpv). At the end of the study, piglets and sows were euthanized following procedures according to the European Directive, using a pentobarbital overdose of 60–100 mg/kg of weight, administered via the jugular vein. Piglets were also euthanized before the end of the trial for animal welfare reasons if they reached a clinical score of 5. The experiment was carried out according to existing Spanish and European regulations and was approved by the Ethical Committee of the Generalitat de Catalonia, Spain under the animal experimentation project number 11970, approved on 12 January 2023.

### 2.3 Detection of CSFV RNA by RT-qPCR

Viral RNA was extracted from serum, nasal, and rectal swabs using the MagAttract 96 cador Pathogen Kit (Qiagen, Hilden, Germany) according to the manufacturer’s instructions. In all cases, RNA was extracted from an initial sample volume of 200 μL to obtain a final volume of 100 μL of RNA, which was stored at −80°C. The presence of CSFV RNA in all samples was analyzed by real time RT-qPCR assay described by Hoffman et al., 2005 (Hoffmann et al., 2005). Reactions were performed using the AgPath-ID™ One-Step RT-PCR Reagents (applied biosystems, Waltham, MA, USA) in an Applied Biosystems^®^ 7500 Fast Real-Time PCR System. Samples were considered positive when the threshold cycle (Ct) value was equal to or less than 40 and negative when fluorescence was undetectable.

### 2.4 Detection of E2-specific CSFV antibodies

The detection of E2-specific antibodies was performed using a commercial ELISA kit (IDEXX Laboratories, Liebfeld, Switzerland). The blocking percentage values were determined following manufacturer’s instruction; values below 30% were considered as negative, between 30 and 40% were considered doubtful, and above 40% were considered as positive.

### 2.5 IFN-α quantification by ELISA test

INF-α levels in serum samples were determined at 7 dpi, using a previously described in-house ELISA test (Diaz de Arce et al., 1992). Briefly, anti-IFN-α monoclonal antibodies (K9 and K17) and serial dilutions of recombinant IFN-α protein (PBL Biomedical Laboratories, Piscataway, NJ, USA) were employed as a standard in this assay. The optical density of the Standard was used to perform a regression curve and quantify the concentration of IFN-α in serum, with the results being expressed as units/mL.

### 2.6 PBMCs samples for RNA-sequencing

For RNA sequencing, PBMCs were collected from three individual pigs inoculated with the CSFV Catalonia01 strain at 7 dpi and three individual pigs vaccinated with the C-strain at 7 dpv, representing three independent biological replicates per experimental group. A total of 10^7^ cells/mL were used for RNA extraction with the RNeasy Mini Kit (Qiagen GmbH, Hilden, Germany) following the manufacturer’s instructions. The RNA quantity and purity were determined with a NanoDrop ND-1000 spectrophotometer (NanoDrop Technologies, Wilmington, DE, USA), and the RNA integrity was analyzed by the Agilent Bioanalyzer-2100 (Agilent Technologies, Inc., Santa Clara, CA, USA). The RNA samples used for this study were of high quality (RNA integrity number (RIN) > 8).

### 2.7 RNA-sequencing analysis

mRNA libraries were sequenced on Illumina NextSeq platform using paired-end 150 bp reads (Illumina, Inc.; San Diego, CA, USA). RNA-seq library preparation and sequencing were carried out by Microomics (IIB Sant Pau, Barcelona, Spain). For quality control and basic statistics, read-quality were calculated using FastQC (Andrews, 2012) and summarized with MultiQC programs (Ewels et al., 2016) then, adapters were removed using Trimmomatic (Bolger et al., 2014). Paired-end reads were mapped to the Sus scrofa Sscrofa11.1 reference genome obtained from Ensembl using STAR (Dobin et al., 2013). Qualimap (García-Alcalde et al., 2012) was used to perform the quality control of the mapping. Gene counts were estimated using featureCounts (Liao et al., 2014). Differential expression analysis was performed with DESeq2 v1.24.0 (Love et al., 2014) with pairwise comparison of PBMCs transcriptomes from pigs infected with Catalonia01 strain at 7dpi and pigs vaccinated with the C-strain vaccine at 7dpv. Significance was considered after adjusting the raw *p*-values to control the false discovery rate (FDR) at 5% by the Benjamini–Hochberg procedure (Benjamini et al., 2001). Adjusted *p*-value < 0.05 were used as cutoff to define differentially expressed genes (DEGs). The RNA-seq data generated in this study have been deposited in the NCBI Sequence Read Archive (SRA) under BioProject accession number PRJNA1499678.

### 2.8 Pathway enrichment analysis

To facilitate biological interpretation of DESeq2 results, gene set enrichment analysis (GSEA) was performed using the clusterProfiler (Yu et al., 2012) on ranked gene lists, with genes ordered by log_2_ fold change (log_2_FC) to identify coordinated biological programs underlying gene expression changes. For GSEA, significantly enriched pathways were defined using an adjusted *p*-value (padjust), corresponding to the false discovery rate (FDR), with a threshold of < 0.05, and were ranked according to their normalized enrichment score (NES).

In parallel, over-representation analysis (ORA) was conducted using Cytoscape v3.10.4 (Shannon et al., 2003) with the ClueGO plugin (Bindea et al., 2009) on DEGs with an absolute |log_2_FC| ≥ 1, with up- and down-regulated genes analyzed separately. For Cytoscape-based enrichment analysis, a *p*-value ≤ 0.05 and kappa score of 0.4 were used as threshold values. Functional annotation and enrichment analysis for both approaches were based on the Kyoto Encyclopedia of Genes and Genomes (KEGG) and Gene Ontology (GO) databases to identify statistically significant and coordinated biological programs underlying the gene expression changes. To further explore functional relationships among the proteins encoded by selected representative DEGs, a protein–protein interaction (PPI) network was generated using STRING v12 (http://string.embl.de/) (Szklarczyk et al., 2023), with a medium confidence score threshold of 0.4.

### 2.9 RT-qPCR analysis of selected differentially expressed genes

RT-qPCR analysis was performed on selected representative DEGs to assess the concordance of the expression patterns identified by RNA-seq using a different analytical method. The same biological samples used for RNA-seq were analyzed by RT-qPCR. TaqMan probe-based RT-qPCR assays were performed to assess selected DEGs identified by RNA-seq and evaluate the concordance of expression patterns. Selected DEGs, indoleamine 2,3-dioxygenase 1 (*ido1*), granulysin (*gnly*) and cluster of differentiation 83 (*cd83*) were analyzed by RT-qPCR using the Ag Path-ID One-step RT-PCR Kit. Primers and probes were designed for the selected genes with their specific sequences obtained from GenBank (**Table 1**). The amplification was carried out in a total volume of 20 µL containing 0.6 μL of forward and reverse primers (20 μM), 0.2 μL of probe (10 μM), and 4 μL of RNA target (25 ng). The cycling steps for the assay included reverse transcription at 48 °C for 10 min, activation of DNA polymerase at 95 °C for 10 min, and 40 cycles of 97 °C for 2 s and 61 °C for 30 s and were performed by an Applied Biosystems^®^ 7500 Fast Real-Time PCR System. The relative expression values of selected genes were calculated by the 2^−ΔΔCt^ method and normalized against the expression levels of the ß-actin gene (Rao et al., 2013).

**Table 1:** The list of primers and probes used in the RT-qPCR.

Gene	Forward primer	Reverse primer	Probe	Accession number
*ido1*	TGAAGATATCCGCAAGGTCCT	GGTCTTCACACTGTATTGCAT	ACTCTCTGAGAAGCTTGGACTGCC	NM_001246240
*gnly*	TGAGTCTTGTCGGAAGATAATCC	GGTCCTCATGATCTTCTTGCACA	TGGGACCACAGCCCAACGAGG	NM_001278755
*cd83*	AGGCAGTCTGTCTTCTAAGACC	GGAAGTTCCCCTGAGTCTATT	TAACCACACTGATGGCTCCTCTGC	XM_021100200
*β-actin*	ATCATGAAGTGCGACGTCGA	TGATCTTCATCGTGCTGGG	TCTACGCCAACAACACGGTGC	(16)

## 3 Results

### 3.1 Absence of clinical signs and detectable humoral response 7 days after Catalonia01 infection and C-strain vaccination

Following Catalonia01 infection and C-strain vaccination of piglets, no clinical signs were observed in any piglet during the first 7 days post infection or vaccination. Likewise, anti-E2 antibodies were undetectable in all animals after 7 days (data not shown).

### 3.2 Higher CSFV RNA detection at 7 dpi in Catalonia01-infected piglets

After 7 days of Catalonia01 infection, all piglets showed moderate levels of CSFV RNA loads in serum samples (Ct= 24.1 - 28.2), except for one piglet which showed a higher viral RNA load (Ct= 21.6). In nasal swabs, CSFV RNA was detected at moderate RNA loads in 25 out of 30 piglets (Ct= 23.9 - 28.5), whereas the remaining 5 piglets showed low RNA loads (Ct ≥ 29.3). Similarly, in rectal swabs, 22 out of 30 piglets showed moderate CSFV RNA loads (Ct =23.7 - 28.9), while the remaining 8 piglets showed low RNA loads (Ct > 29.6). In contrast, at 7 days after vaccination with the C-strain vaccine, CSFV RNA was detected in only a limited number of piglets. Among the 15 vaccinated piglets, CSFV RNA was detected in the serum of three piglets, in the nasal swabs of one piglet, and in the rectal swab of another piglet, with low RNA loads in all cases (Ct ≥ 35.5) (**Figure 1**).

**Figure 1:**
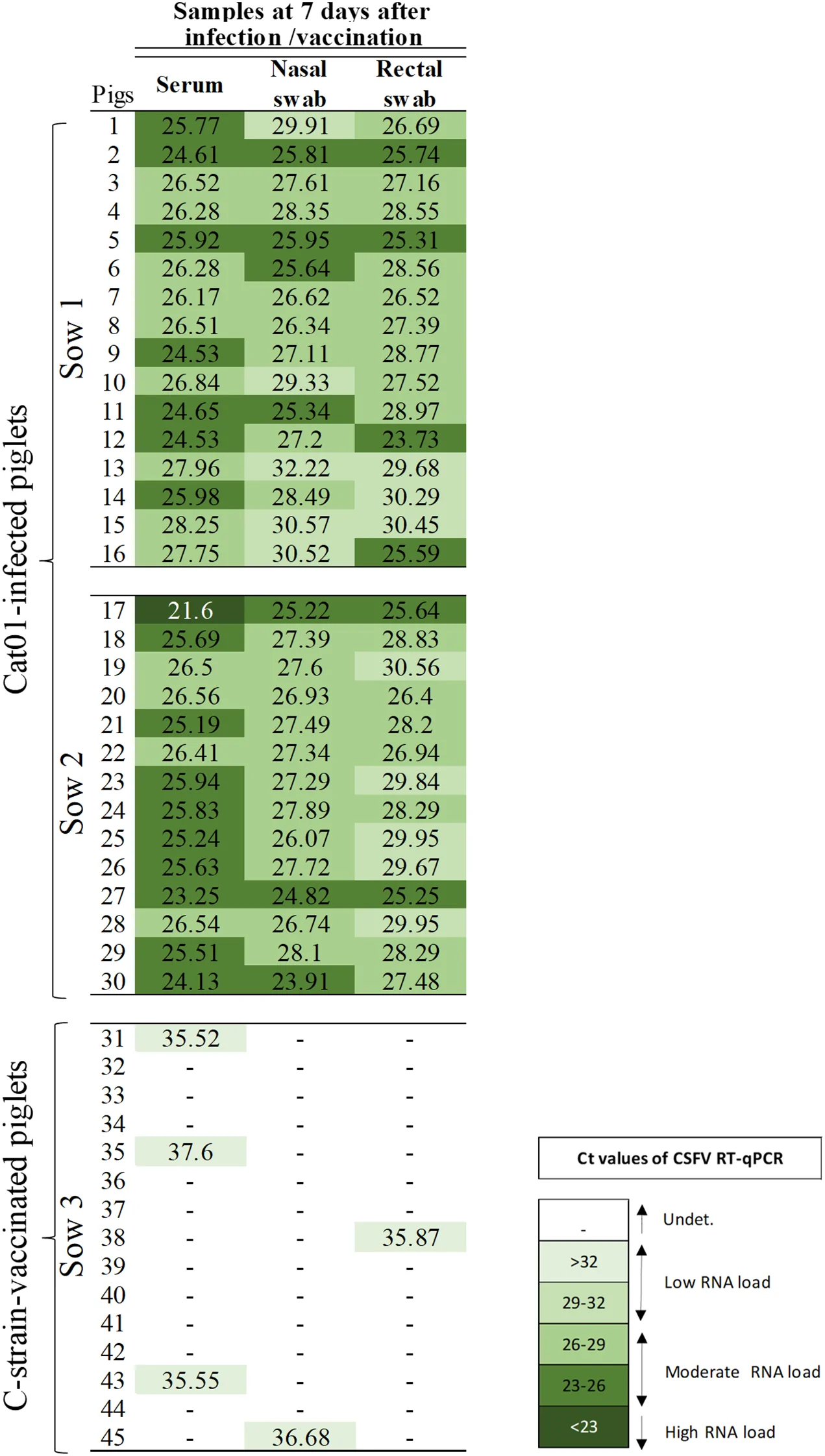
CSFV RNA detection in serum, nasal, and rectal swab samples from piglets infected with the Catalonia01 strain or vaccinated with C-strain at 7 days post-infection/post-vaccination (dpi/dpv), determined by RT-qPCR. Viral RNA load is represented by color intensity and categorized according to Ct values as low (Ct > 29), moderate (Ct = 23–29), or high (Ct < 23). Ct values > 40 were considered negative.

### 3.3 Increased serum IFN-α levels at 7 dpi in Catalonia01-infected piglets

A stronger type I interferon response, measured as serum IFN-α concentration, was observed in piglets infected with the Catalonia01 strain compared to C-strain–vaccinated piglets at 7 dpi/dpv. IFN-α levels in Catalonia01-infected animals ranged from 200 to 480 units/mL and were significantly higher than those detected in C-strain–vaccinated piglets, which ranged from 40 to 75 units/mL (**Figure 2**).

**Figure 2:**
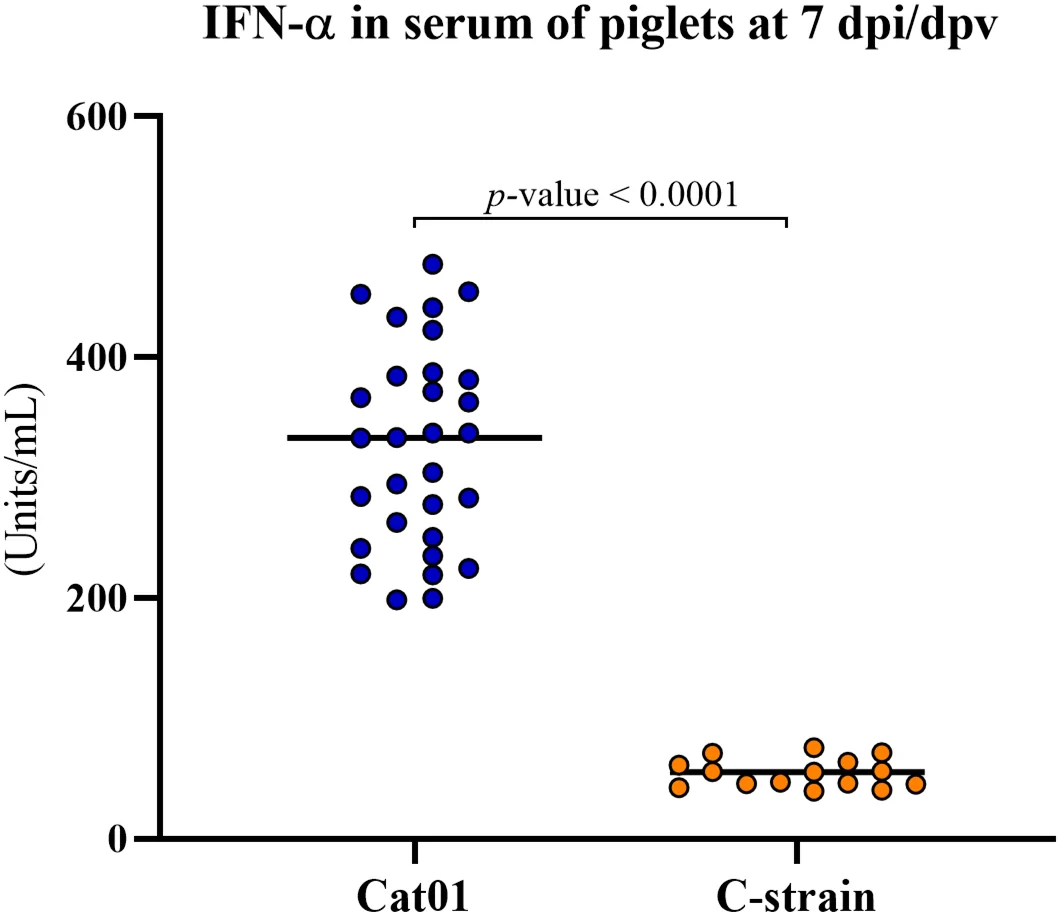
IFN-α level in serum of piglets infected with Catalonia01 strain (blue circles) at 7 days post infection (dpi) or vaccinated with C-strain (orange circles) at 7 days post-vaccination (dpv) measured by ELISA. The results are expressed in units/mL. Mann Whitney test was performed to statistically compared the two groups.

### 3.4 Differential gene expression and functional pathway enrichment in PBMCs from Catalonia01-infected piglets

Differential gene expression analysis was performed on PBMCs from Catalonia01-infected piglets at 7 dpi and C-strain–vaccinated piglets at 7 dpv. Among the 15,271 expressed genes detected, 616 were differentially expressed genes (DEGs; adjusted *p*-value ≤ 0.05), of which 209 were downregulated and 407 upregulated genes in Catalonia01-infected piglets compared to C-strain-vaccinated piglets. The overall distribution of DEGs and the relationship between gene expression levels and log_2_FC are shown in the volcano and MA plots (**Supplementary Figure S1**).

For Gene Set Enrichment Analysis (GSEA), all expressed genes (n = 15,271) were ranked according to their log_2_FC values in decreasing order, and the resulting ranked list was used to identify significantly enriched pathways (*p*-adjust ≤ 0.05). Using the GO Biological Process database, neutrophil migration was identified as the most significantly enriched pathway in PBMCs from Catalonia01-infected piglets while pathways related to the extracellular matrix and structure organization, external encapsulating structure organization, regulation of cilium movement involved in cell motility and regulation of cilium-dependent cell motility were significantly negatively enriched compared with C-strain-vaccinated piglets (**Figure 3A**). GSEA identified significant positive enrichment of KEGG pathways involved in natural killer cell-mediated cytotoxicity, JAK-STAT signaling, linoleic acid metabolism, neuroactive ligand-receptor interaction and cytokine-cytokine receptor interaction in PBMCs from Catalonia01-infected piglets. In contrast, pathways related to parathyroid hormone synthesis, secretion and action, melanogenesis, proteoglycans in cancer, ribosome and protein digestion and absorption were significantly negatively enriched compared with C-strain-vaccinated piglets (**Figure 3B**).

**Figure 3:**
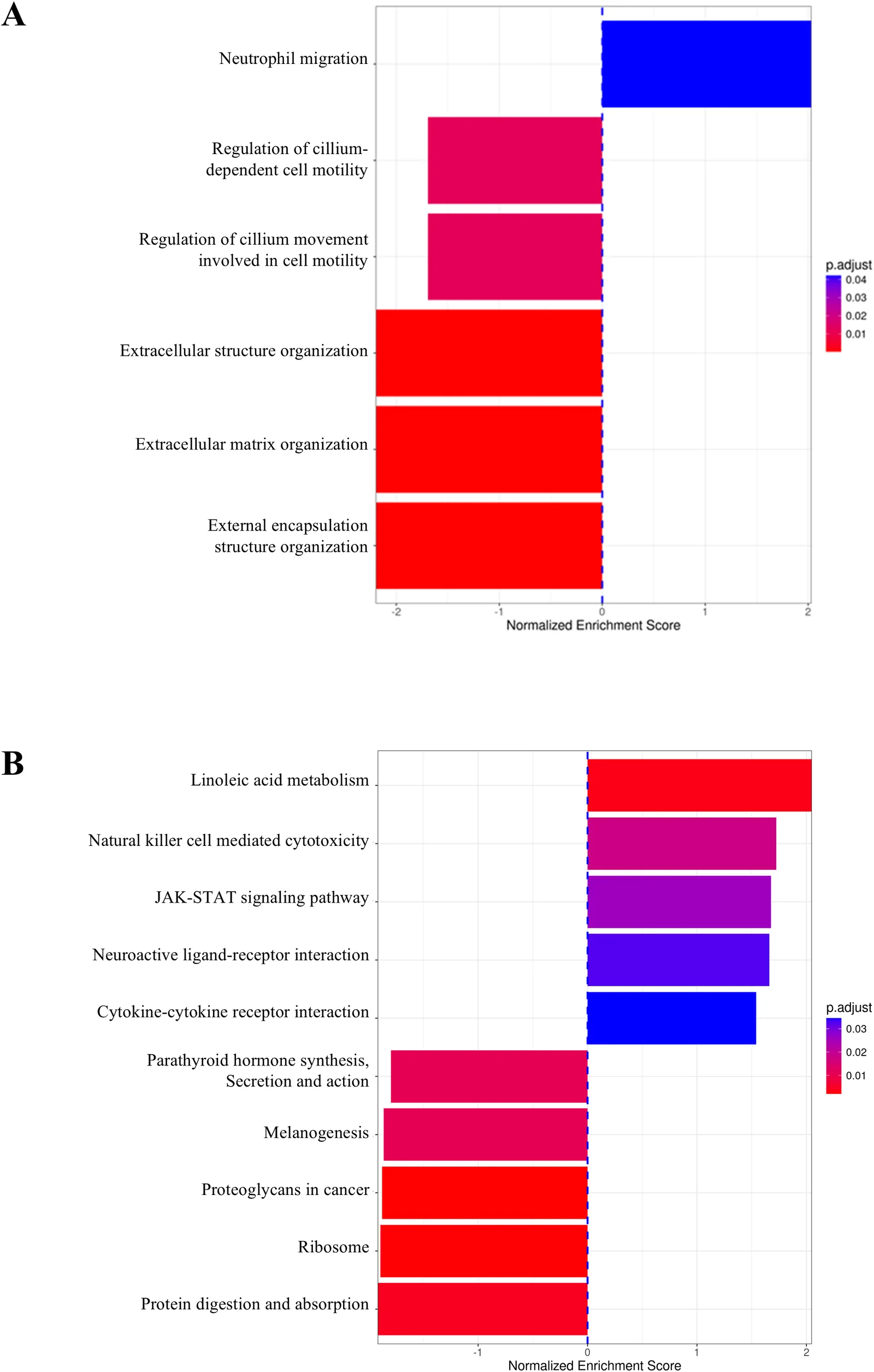
Gene set enrichment analysis (GSEA) comparing PBMCs from Catalonia01-infected piglets at 7 dpi and C-strain–vaccinated piglets at 7 dpv. **(A)** GO Biological Process terms and **(B)** KEGG pathways are ranked by normalized enrichment score (NES). Positive NES values indicate pathways enriched in Catalonia01-infected piglets, whereas negative NES values indicate pathways enriched in C-strain–vaccinated piglets. Colour intensity indicates the level of statistical significance, represented by the adjusted *p*-value (FDR).

To further characterize the functional implications of gene expression changes in PBMCs associated with Catalonia01 infection compared with C-strain vaccination, over-representation analysis (ORA) of DEGs was performed. Functional enrichment analysis integrating GO and KEGG annotations revealed overrepresented functional categories among upregulated DEGs, including cytokine-mediated signaling pathway, lymphocyte mediated immunity, positive regulation of ion transport, regulation of chemotaxis, graft-versus-host disease, negative regulation of lipid biosynthetic process, plasma membrane bounded cell projection cytoplasm, amoebiasis, cyclic-nucleotide-mediated signaling and eye photoreceptor cell differentiation (**Figure 4A**). Conversely, overrepresented functional categories among downregulated DEGs included MHC class II protein complex, cell-cell junction organization, extracellular matrix organization, glycosphingolipid biosynthesis, salivary secretion, ether lipid metabolism, positive regulation of protein localization to plasma membrane, cellular response to metal ion, regulation of endothelial cell migration, positive regulation of JUN kinase activity and B cell homeostasis (**Figure 4B**).

**Figure 4:**
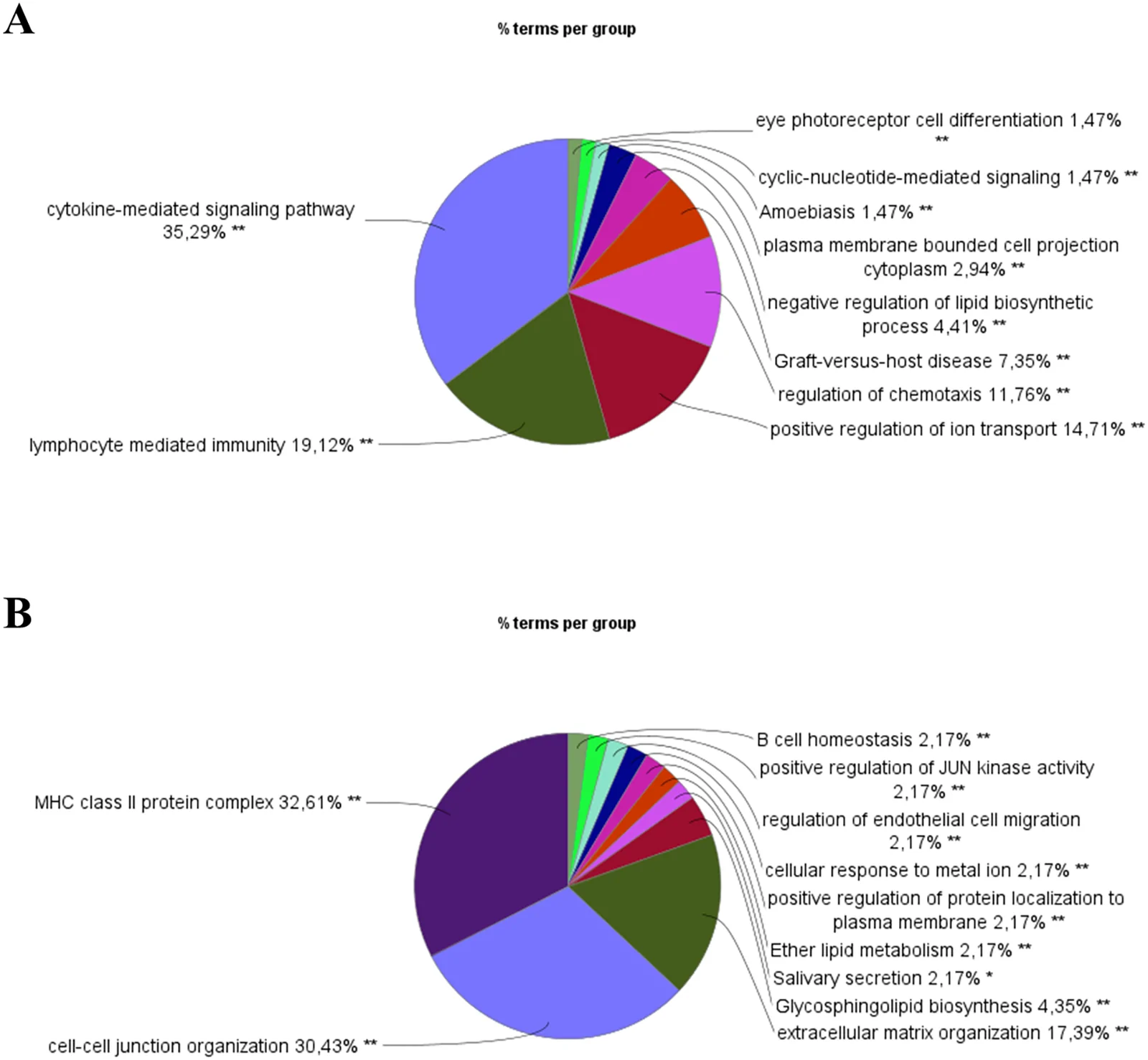
Over-representation analysis (ORA) of significantly differentially expressed genes (DEGs; adjusted *p* < 0.05) in PBMCs from Catalonia01-infected piglets compared with C-strain-vaccinated animals. Functional enrichment analysis was performed using GO terms and KEGG pathways. The multilevel pie charts show enriched **(A)** upregulated and **(B)** downregulated functional terms in PBMCs from Catalonia01-infected piglets at 7 dpi. Significantly enriched terms were identified using ClueGO within Cytoscape (*p* ≤ 0.05; kappa score = 0.4). Asterisks indicate the level of statistical significance (**p < 0.01).

### 3.5 Representative immune signatures associated with early persistent CSFV infection

To facilitate the biological interpretation of transcriptomic data, representative genes were selected to illustrate the major immune pathways identified by ORA and GSEA (**Figure 5A**). Based on GO and KEGG annotations together with their established biological functions described in the literature, genes were assigned to three functional categories. Genes associated with cytotoxic effector responses included *gnly*, interleukin 12 receptor subunit beta 2 (*il12rb2*), Fas ligand (*faslg*), granzyme B (*gzmb*), natural cytotoxicity triggering receptor 1 (*ncr1*), perforin 1 (*prf1*), T-box transcription factor 21 (*tbx21****)***, natural killer cell granule protein 7 (*nkg7*) and RUNX family transcription factor 3 (*runx3*). Immune regulation was represented by lymphocyte activating gene 3 (*lag3*) and *ido1*, whereas antigen presentation included SLA-DQ beta1 (*sla-dqb1*), MHC class II histocompatibility antigen SLA-DQA (*sla-dqa1*), MHC class II, DM beta (*sla-dmb*) and *cd83*. Genes were assigned to each functional category based on GO and KEGG annotations together with published evidence supporting their biological roles.

**Figure 5:**
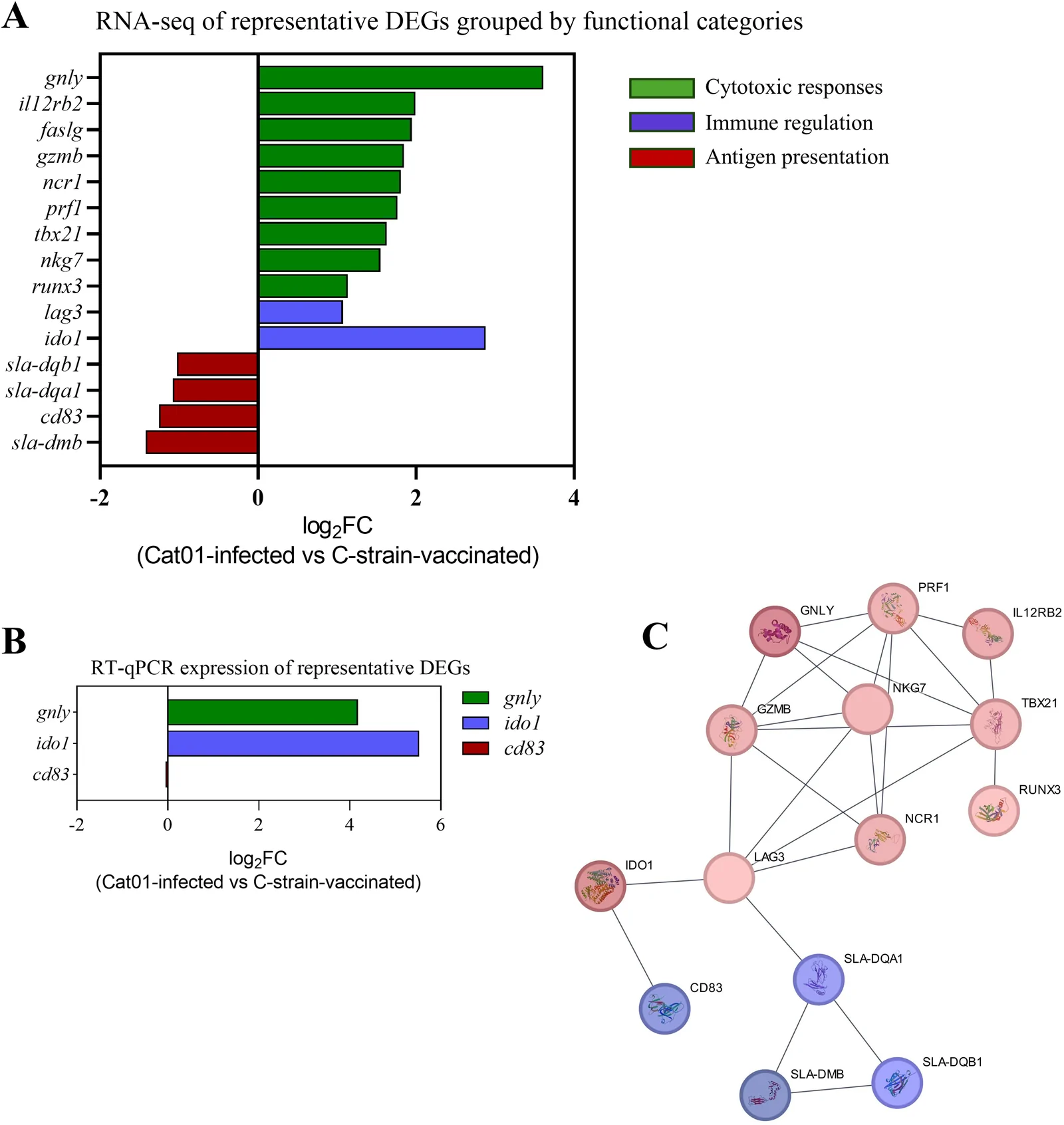
**(A)** RNA-seq and **(B)** RT-qPCR expression of representative differentially expressed genes (DEGs) grouped according to their biological functions. Bars represent log2 fold change (log_2_FC) in PBMCs from Catalonia01-infected piglets relative to C-strain-vaccinated piglets at 7 days post-infection/vaccination. Genes are grouped into cytotoxic effector response (green), immune regulation (blue), and antigen presentation (red). RT-qPCR log_2_FC values were calculated using the ΔΔCt method with β-actin as the endogenous control. **(C)** Protein–protein interaction (PPI) network generated using STRING based on proteins encoded by representative DEGs. Nodes represent proteins, whereas edges indicate known or predicted functional associations. Node colors indicate upregulated (red) and downregulated (blue) proteins corresponding to DEGs identified in Catalonia01-infected piglets.

Consistent with these transcriptomic signatures, RT-qPCR analysis of the representative genes *gnly*, *ido1*, and *cd83* reproduced the expression patterns identified by RNA-seq. *gnly* and *ido1*, two of the most upregulated DEGs in RNAseq, representatives of the cytotoxic and immunoregulatory responses, respectively, were markedly upregulated by RT-qPCR analysis, whereas *cd83*, associated with antigen presentation, showed a moderate reduction in expression in Catalonia01-infected piglets compared with C-strain-vaccinated animals (**Figure 5B**).

STRING analysis of proteins encoded by the selected representative DEGs revealed a highly interconnected PPI network comprising 14 nodes and 25 edges (PPI enrichment *p* < 1 × 10^-16^), indicating functional associations among proteins involved in cytotoxic effector responses, immune regulation, and antigen presentation (**Figure 5C**).

## 4 Discussion

The present study was designed to compare two divergent early outcomes following CSFV exposure: Catalonia01 infection leading to postnatal persistence and protective immunization with the C-strain vaccine. Within this comparative framework, the transcriptional differences identified at 7 dpi/dpv reflect the relative expression profiles associated with these two immune states. The results presented here provide a basis for exploring the distinct immune responses associated with these divergent outcomes.

Understanding the earliest host immune events associated with persistent CSFV infection remains one of the major unresolved questions in CSF pathogenesis. In the present study, we compared the early transcriptional profiles of PBMCs from neonatal piglets infected with the moderately virulent Catalonia01 strain, previously characterized by its capacity to induce postnatal persistent infection (Muñoz-González et al., 2015b), with those from piglets vaccinated with the live attenuated C-strain vaccine. Importantly, analyses were performed at 7 days post infection or vaccination, before the appearance of clinical signs or detectable anti-CSFV antibodies. Consistent with the phenotype previously described for postnatally persistently infected piglets (Muñoz-González et al., 2015b), Catalonia01-infected animals already exhibited high viral RNA loads in serum and secretions together with increased serum IFN-α concentrations, demonstrating that active systemic viral replication was established despite the absence of clinical manifestations or detectable humoral responses.

Type I interferons are among the earliest antiviral mediators induced following CSFV infection and are primarily produced by plasmacytoid dendritic cells (pDCs) (Mair et al., 2022), which can subsequently promote activation of innate immune populations such as NK and γδ T cells during the early stages of infection (Franzoni et al., 2014). Previous transcriptomic studies demonstrated strong activation of interferon-driven pathways in CSFV-infected pDCs, particularly following infection with virulent strains (Auray et al., 2020). Consistent with these observations, our study revealed markedly increased serum IFN-α levels together with enrichment of JAK–STAT and cytokine signaling pathways in PBMCs from Catalonia01-infected piglets, indicating that even moderately virulent strains capable of establishing persistence can trigger potent innate antiviral responses during the earliest stages of infection.

In line with this strong innate antiviral activation, transcriptomic profiling revealed a pronounced cytotoxic transcriptional signature in PBMCs from Catalonia01-infected piglets compared with C-strain-vaccinated animals. One of the strongest findings of this study was the marked upregulation of genes associated with cytotoxic effector responses, including *gnly, prf1, gzmb* and *faslg*, which constitute key components of the cytolytic machinery used by cytotoxic T lymphocytes and NK cells to eliminate infected cells (Lagumdzic et al., 2022). Increased expression of *nkg7*, a marker associated with cytotoxic granule release (Turiello et al., 2025), together with transcriptional regulators such as *tbx21* and *runx3* (Cruz-Guilloty et al., 2009), further supports the cytotoxic transcriptional profile observed in Catalonia01-infected piglets, consistent with a type 1-polarized antiviral response. Notably, *runx3* plays a key role in cytotoxic CD8^+^ T-cell differentiation and in the regulation of effector molecules such as *prf1* and *gzmb* (Cruz-Guilloty et al., 2009). Consistent with our findings, a similar transcriptional signature has been described in differentiated porcine CD8^+^ T-cell subsets, in which genes involved in cytolytic activity, including *gnly, prf1, gzmb, faslg, tbx21* and *runx3* are highly expressed in porcine intermediate and terminally differentiated CD8^+^ T cells (Lagumdzic et al., 2022). In addition, the upregulation of *ncr1* is consistent with the enhanced cytotoxic profile observed in our study, given that ncr1 is a member of the natural cytotoxicity receptor family, which plays a central role in activating cytotoxic effector functions (Mair et al., 2016; Pazina et al., 2017). The upregulation of *il12rb2* further supports the enhanced cytotoxic signature identified in this study, as *il12rb2* encodes the signaling subunit of the high-affinity IL-12 receptor, which promotes cytotoxic effector functions and IFN-γ production in NK and CD8^+^ T cells (Zhang et al., 2008; Hwang et al., 2023). Previous studies investigating cellular immune responses to CSFV demonstrated that moderately virulent strains can induce IFN-γ-producing CD4^-^CD8β^+^ lymphocytes expressing perforin and cytotoxic mediators, supporting the activation of cytotoxic immune programs during infection (Summerfield and Ruggli, 2015). Together, these findings indicate that Catalonia01 infection is associated with an early cytotoxic transcriptional profile, highlighting the engagement of cell-mediated antiviral responses during the first week of infection. Importantly, this transcriptional signature was already detectable before clinical manifestations or seroconversion became evident. As these transcriptional profiles were obtained from bulk PBMCs, they reflect the integrated response of the circulating immune compartment and may result from both transcriptional regulation and differences in the relative abundance of immune cell populations. Future studies incorporating immune cell phenotyping or single-cell transcriptomic approaches will be necessary to resolve the cellular origin of these transcriptional patterns. However, the marked cytotoxic profile and the strong type I interferon signature identified in infected piglets do not appear to reflect an efficient antiviral response capable of controlling viral replication. Despite the absence of clinical signs, infected animals maintained extremely high viral RNA loads in blood and secretions, consistent with previous reports describing postnatally persistently infected piglets as highly efficient viral shedders (Muñoz-González et al., 2015b). Thus, the transcriptional profile observed at 7 dpi suggests the coexistence of antiviral and immunoregulatory signatures in the absence of effective viral clearance, which ultimately may favor the establishment of persistence. This apparent dissociation between immune activation and viral control is consistent with features of early dysregulated immune state that does not result in effective viral clearance. Our findings are consistent with the well-established concept that persistent viral infections promote sustained inflammatory signaling, chronic antigenic stimulation, and activation of immunoregulatory pathways, thereby progressively impairing antiviral immunity (Virgin et al., 2009; Zuniga et al., 2015; Collier et al., 2021). Accordingly, persistent type I interferon signaling has been associated with immune dysregulation, lymphocyte dysfunction, and viral persistence rather than effective viral control (Snell et al., 2017).

At 7 dpi, Catalonia01-infected piglets displayed markedly increased IFN-α levels together with enrichment of JAK–STAT and cytotoxic antiviral pathways, indicating a robust early antiviral response. Nevertheless, the persistence of high systemic viral loads despite this antiviral signature suggests that additional immunoregulatory mechanisms may already be operating during the earliest stages of infection. Indeed, the concurrent upregulation of immunoregulatory genes such as *lag3* and *ido1* may suggest the early establishment of inhibitory mechanisms that may constrain the antiviral activity of cytotoxic lymphocytes. Both *lag3* and *ido1* are key components of immunoregulatory pathways known to suppress T-cell activity during persistent viral infections (Wilson et al., 2012). In particular, *lag3* has an inhibitory role in regulating CD8(+) T cells during chronic virus infection (Cook and Whitmire, 2016), whereas *ido1* contributes to immune regulation through tryptophan catabolism. Consistent with this role, CSFV-induced *ido1* activity suppresses NF-κB signaling and antiviral cytokine responses, further supporting its involvement in modulating antiviral immunity (Zhao et al., 2024). Moreover, the increased expression of *ido1* observed in Catalonia01-infected piglets is consistent with activation of interferon-driven immunoregulatory pathways, as previously reported in chronic viral infections (Snell et al., 2017). Through tryptophan catabolism, *ido1* contributes to the suppression of T-cell responses and the establishment of immune tolerance, undersconring its role as a key regulator of antiviral immunity (Schmidt and Schultze, 2014; Lepiller et al., 2015).

Therefore, the concurrent upregulation of *lag3* and *ido1* in Catalonia01-infected piglets supports the coexistence of early antiviral/cytotoxic immune activation and immunoregulatory pathways that may limit effective T-cell responses and contribute to incomplete viral clearance during persistent CSFV infection, although the functional consequences of these patterns remain to be established. Although type I interferon responses are essential for early antiviral defense, the simultaneous induction of these immunoregulatory markers, together with a comparatively lower MHC class II-associated transcriptional profile, suggests that this early antiviral activation may coexist with regulatory features potentially limiting the development of an effective antiviral response.

In contrast, C-strain-vaccinated piglets exhibited lower IFN-α levels at this time point and a distinct transcriptional profile characterized by enrichment of the MHC class II protein complex pathway, consistent with a stronger antigen-presentation-associated transcriptional profile and early adaptive immune priming. Although vaccinated animals remained seronegative at 7 dpv, transcriptomic analysis revealed a profile associated with antigen presentation and adaptive immune development. Conversely, the lower expression of MHC class II-associated genes in Catalonia01-infected relative to C-strain-vaccinated piglets may reflect differences in antigen-presentation-associated responses and subsequent adaptive immune development, potentially contributing to the absence of antibody responses previously described in CSFV persistently infected pigs (Muñoz-González et al., 2015b; Ganges et al., 2020). Previous studies have shown that CSFV infect dendritic cells without significantly altering the expression of MHC molecules or co-stimulatory markers (Carrasco et al., 2004), indicating that the comparatively lower MHC class II signature observed here is unlikely to result from a direct viral effect on antigen-presenting cells. Instead, it may reflect broader alterations in antigen presentation, immune cell composition, or systemic immune activation, consistent with the immune dysregulation and defective coordination between innate and adaptive immunity described in chronic viral infections and tolerogenic immune environment across multiple species (Zuniga et al., 2015; Collier et al., 2021; Paludan et al., 2021). In parallel, C-strain vaccinated piglets also showed upregulation of genes involved in extracellular matrix organization, cytoskeletal regulation, and protein localization when comparing with Catalonia01-infected piglets. Although not directly linked to antiviral immunity, these transcriptional changes may reflect early molecular adaptations accompanying vaccine-induced immune activation. These transcriptional findings are particularly relevant because both groups, Catalonia01-infected and C-strain vaccinated, remained clinically healthy and seronegative at 7 dpi, indicating that distinct transcriptional programs are already detectable during the earliest stages of moderately virulent Catalonia01 infection and live attenuated C-strain vaccination, well before conventional immunological or clinical markers become detectable.

The present study also highlights the potential value of transcriptomic approaches for identifying genes of interest associated with the early response to persistent CSFV infection. Among the differentially expressed genes, *gnly* emerged as one of the most strongly upregulated transcripts in Catalonia01-infected piglets. Importantly, the expression pattern of *gnly* identified by RNA-seq was also reproduced by RT-qPCR, supporting the consistency of this finding across the two analytical methods. Overall, the transcriptional patterns identified in this study highlight a set of genes of interest associated with the early response to Catalonia01 infection, whose broader relevance as potential markers will require further evaluation in independent cohorts. Together, cytotoxicity-associated genes such as *gnly*, along with immunoregulatory genes such as *ido1*, are part of a distinct transcriptional signature associated with Catalonia01 infection compared with C-strain vaccination before the onset of clinical signs or seroconversion. Further investigation of these transcriptional patterns may improve our understanding of the early host responses associated with CSFV persistence, which is particularly relevant given the role of persistently infected animals as clinically silent reservoirs that may escape conventional serological surveillance (Coronado et al., 2019; Ganges et al., 2020). STRING analysis of proteins encoded by representative DEGs further highlighted functional associations among proteins involved in antigen presentation, cytotoxic effector mechanisms, and immune regulation. Although interactions between upregulated and downregulated proteins do not imply coordinated expression, these functional networks support the coexistence of cytotoxic transcriptional programs with comparatively lower expression of MHC class II-associated genes in Catalonia01-infected piglets, reinforcing the concept of an early but differently regulated antiviral immune response.

Collectively, our findings reveal an early transcriptional profile associated with postnatal persistent CSFV infection characterized by the coexistence of antiviral/cytotoxic responses and immunoregulatory signatures in the absence of effective viral clearance. Following Catalonia01 infection, high viral replication was accompanied by increased systemic IFN-α, enrichment of JAK–STAT-related pathways, and increased expression of cytotoxic effector genes such as *gnly, prf1, gzmb*, and *faslg*. In the context of previous studies identifying plasmacytoid dendritic cells as a major source of type I interferons during CSFV infection and linking these cytotoxic effector molecules to NK- and CD8^+^ T-cell responses, these findings are consistent with the early engagement of both interferon-mediated antiviral signaling and cytotoxic immune responses. However, rather than progressing toward efficient viral clearance, this early antiviral response coexisted with increased expression of the immunoregulatory genes *lag3* and *ido1*, together with the previously described expansion of suppressive 6D10^+^ granulocytic cell populations displaying MDSC-like characteristics (Bohorquez et al., 2019). Together, these observations suggest that immunoregulatory processes may be associated with failure to achieve effective viral clearance, although their functional contribution to CSFV persistence remains to be established.

In parallel, the comparatively lower expression of MHC class II-associated genes in Catalonia01-infected animals may reflect differences in antigen-presentation-associated responses that could influence subsequent CD4^+^ T-cell priming and the development of effective humoral immunity. Under this scenario, infected animals would enter a state of sustained but dysregulated immune activation characterized by continuous viral replication, persistent antigenic stimulation, and cytotoxic responses that fail to eliminate the virus. Although the functional mechanisms underlying this scenario were not directly investigated in the present study, such an immune configuration may favor long-term viral persistence while limiting excessive immunopathology and could contribute to the clinically silent phenotype observed in persistently infected animals despite sustained systemic infection and high viral load. In contrast, vaccination with the attenuated C-strain was associated with lower viral replication, a stronger MHC class II-associated transcriptional profile, and a more coordinated immune response consistent with the progressive development of protective adaptive immunity. Overall, this model would resemble the immune adaptations described in other persistent viral infections, in which chronic antigen stimulation promotes a regulated state of immune dysfunction that simultaneously limits viral clearance and excessive tissue damage, thereby favoring long-term host survival and continued viral transmission.

Taken together, our findings show that early postnatal persistent infection with the moderately virulent Catalonia01 CSFV strain is established in the context of high systemic viral replication and a marked antiviral transcriptional response, while infected piglets remain clinically healthy and seronegative during the earliest stages of infection. By further characterizing the early host response associated with CSFV persistence, this study identifies transcriptional differences between Catalonia01 infection and C-strain vaccination that provide a basis for future investigation of their potential relevance as molecular markers of persistent infection. Given the critical role of persistently infected animals as silent reservoirs of virus transmission, a better understanding of these early host–virus interactions may contribute to improving surveillance strategies and disease control in regions where classical swine fever remains endemic. More broadly, our findings expand on previous evidence regarding the immune response during persistent CSFV infection by providing a more detailed characterization of a distinct early transcriptional profile associated with the establishment of persistence compared with the protective response induced by vaccination. This profile is characterized by the coexistence of antiviral/cytotoxic and immunoregulatory signatures in the absence of effective viral clearance. Similar patterns of concurrent antiviral and immunoregulatory responses have been described in persistent viral infections in other species, suggesting that the findings reported here may have broader relevance for understanding host responses associated with viral persistence.

## Data Availability

The datasets presented in this study can be found in online repositories. The names of the repository/repositories and accession number(s) can be found in the article.
